# Using Social Media to Engage and Enroll Underrepresented Populations: Longitudinal Digital Health Research

**DOI:** 10.2196/68093

**Published:** 2025-04-15

**Authors:** Christiana Harry, Sarah Goodday, Carol Chapman, Emma Karlin, April Joy Damian, Alexa Brooks, Adrien Boch, Nelly Lugo, Rebecca McMillan, Jonell Tempero, Ella Swanson, Shannon Peabody, Diane McKenzie, Stephen Friend

**Affiliations:** 1 4YouandMe Great Neck, NY United States; 2 Department of Psychiatry University of Oxford Oxford United Kingdom; 3 Crohn’s and Colitis Foundation New York, NY United States; 4 Moses/Weitzman Health System Middletown, CT United States; 5 Evidation Health San Mateo, CA United States; 6 Washington University St. Louis, MO United States; 7 University of California San Diego Health San Diego, CA United States; 8 RespirAI US Inc Omaha, NE United States; 9 Dana-Farber Cancer Instituite Boston, MA United States

**Keywords:** digital health research, digital health technology, recruitment, research subject, participant, pregnancy, maternal health, underrepresented populations, health equity, diversity, marginalized, advertisement, social media, retention, attrition, dropout

## Abstract

**Background:**

Emerging digital health research poses roadblocks to the inclusion of historically marginalized populations in research. Exclusion of underresourced communities in digital health research is a result of multiple factors (eg, limited technology access, decreased digital literacy, language barriers, and historical mistrust of research and research institutions). Alternative methods of access and engagement may aid in achieving long-term sustainability of diversified participation in digital health research, ensuring that developed technologies and research outcomes are effective and equitable.

**Objective:**

This study aims to (1) characterize socioeconomic and demographic differences in individuals who enrolled and engaged with different remote, digital, and traditional recruitment methods in a digital health pregnancy study and (2) determine whether social media outreach is an efficient way of recruiting and retaining specific underrepresented populations (URPs) in digital health research.

**Methods:**

The Better Understanding the Metamorphosis of Pregnancy (BUMP) study was used as a case example. This is a prospective, observational, cohort study using digital health technology to increase understanding of pregnancy among 524 women, aged 18-40 years, in the United States. The study used different recruitment strategies: patient portal for genetic testing results, paid/unpaid social media ads, and a community health organization providing care to pregnant women (Moses/Weitzman Health System).

**Results:**

Social media as a recruitment tool to engage URPs in a digital health study was overall effective, with a 23.6% (140/594) enrollment rate of those completing study interest forms across 25 weeks. Community-based partnerships were less successful, however, resulting in 53.3% (57/107) engagement with recruitment material and only 8.8% (5/57) ultimately enrolling in the study. Paid social media ads provided access to and enrollment of a diverse potential participant pool of race- or ethnicity-based URPs in comparison to other digital recruitment channels. Of those that engaged with study materials, paid recruitment had the highest percentage of non-White (non-Hispanic) respondents (85/321, 26.5%), in comparison to unpaid ads (Facebook and Reddit; 37/167, 22.2%). Of the enrolled participants, paid ads also had the highest percentage of non-White (non-Hispanic) participants (14/70, 20%), compared to unpaid ads (8/52, 15.4%) and genetic testing service subscribers (72/384, 18.8%). Recruitment completed via paid ads (Instagram) had the highest study retention rate (52/70, 74.3%) across outreach methods, whereas recruitment via community-based partnerships had the lowest (2/5, 40%). Retention of non-White (non-Hispanic) participants was low across recruitment methods: paid (8/52, 15.4%), unpaid (3/35, 14.3%), and genetic testing service subscribers (50/281, 17.8%).

**Conclusions:**

Social media recruitment (paid/unpaid) provides access to URPs and facilitates sustained retention similar to other methods, but with varying strengths and weaknesses. URPs showed lower retention rates than their White counterparts across outreach methods. Community-based recruitment showed lower engagement, enrollment, and retention. These findings highlight social media’s potential for URP engagement and enrollment, illuminate potential roadblocks of traditional methods, and underscore the need for tailored research to improve URP enrollment and retention.

## Introduction

### Background

Participation in research studies by historically racial and ethnic marginalized populations is significantly lower compared to their White counterparts. This has resulted in the underrepresentation of these groups across research study disciplines [1-3]. The National Institutes of Health (NIH) defines underrepresented populations (URPs) as those with disproportionately low representation relative to their overall or disease-specific population [4]. Despite mandates to include these groups in federally funded research, factors such as general attitudes toward research, sociocultural barriers, and accessibility continue to hinder diversity in research populations [1,5-11].

Certain URPs, such as African American populations, face additional historical and structural barriers to research participation including low health literacy, lack of access to care, and mistrust of institutions [2]. For URPs, particularly African American populations, mistrust of academic and research institutions is the largest reported participation barrier due to historical events, such as the Tuskegee syphilis study, and worsened by present inequalities in the health care system [1]. As a direct result of this severe institutional mistrust among URPs generally, lack of engagement and subsequent enrollment of URPs in research studies persists and continues to contribute to health disparities within these specific population subgroups, as they tend to reject participatory outreach from more traditional recruitment methods associated with these mistrusted institutions and present health inequities [1].

These health inequities are especially seen in pregnant populations, as illustrated by present maternal health outcome statistics [12]. Since 2019, the maternal mortality rate in the United States has continued to rise steadily, jumping to 32.9 deaths per 100,000 live births in 2021, compared with a rate of 20.1 in 2019 [12]. For pregnant women of ethnic or racial URPs, this discrepancy in maternal health outcomes is hypervisible. The historical exclusion of pregnant women from research studies has been present due to a perceived concern of lack of direct participant benefit or risk of significant harm to offspring. This exclusion has resulted in gaps in scientific knowledge regarding key women’s health issues that further exacerbate women’s health outcome disparities [13].

As these disparities and roadblocks to participation continue to be observed, additional efforts are also necessary to identify successful evidence-based enrollment strategies for reaching and engaging URPs, especially in digital research studies. Current engagement and recruitment strategies of URPs often emphasize outreach via community-forward channels (eg, partnerships with community leaders, churches, community-based clinic sites, and other organizations) to alleviate some of the barriers of mistrust present in these communities [10]. However, conflicting evidence exists regarding the effectiveness of community outreach as an isolated strategy for increasing the enrollment of URPs [10].

Another suggested recruitment strategy to bolster the engagement and involvement of URPs in studies is social media advertising. More recently, social media has been used by researchers as a new source of participant recruitment and may be an especially useful tool in locating specific URPs due to the widespread reach of social media to potential participants who otherwise might have been not visible to researchers, the anonymity that social media provides users, and general mistrust of health care institutions and more traditional recruitment methods by URPs [2,14,15].

Over the past decade, social media use in the United States has steadily increased. As of 2022, over 300 million people in the United States use social media, making the United States home to the third largest social media audience globally [16,17]. The social media platforms Facebook (Meta), Twitter (X Corp), and Instagram (Meta) account for a combined 83% of all social media site visits in the United States [16]. As nearly 3 quarters of Americans report social media use, social media platforms now provide researchers a new, accessible alternative to traditional recruitment methods (eg, printed flyers and in-person outreach) to meet individuals where they are and help bridge the digital divide [18].

The digital divide refers to the patterns of difference in the utilization of technology, the internet, and social media by race, ethnicity, or socioeconomic status. The underlying drivers of the digital divide are manifold but include 2 key factors: differential access to and engagement (motivation) in the use of digital health technologies (DHTs; smartphones, wearable devices or other digital products, electronic health records, and telehealth options). The use of social media or DHTs in digital research studies requires ownership of devices (eg, smartphones and wearable devices), adequate internet access, and a level of digital and health literacy, indicating the presence of additional cost and other barriers to digital health engagement and use of DHTs [19].

The other key driver of the digital divide is the disconnect in engagement, especially among URPs. Although social media usage for racially based URPs continues to surpass that of their White counterparts, these populations are significantly less likely to engage with health information on the internet and social media or use social networking channels to learn more about their health [20-22]. However, studies indicate expressed interest by specific URPs (eg, African American women) in using DHTs and social media to engage with health information [23-25].

As the use of DHTs in digital research studies continues to rapidly expand, new and innovative ways in which these technologies may be used to support individual and population health research also evolve [26,27]. DHTs and digital research studies present the potential to mitigate existing gaps in health care access, quality of care, and health care outcomes, further suggesting that social media may be a powerful, untapped resource in reaching and engaging URPs in DHT research studies and digital health [22].

### Previous Work

Existing research studies have primarily used Facebook as a means of recruiting URPs, especially for stigmatized groups, but few research studies explore the feasibility and effectiveness of using other social media platforms and recruitment methods specifically to engage URPs in digital health studies [28]. The use of more nuanced social media outreach methods—such as advertisements on Instagram (a platform that has nearly 127.2 million active users in the United States as of 2023)—to recruit and engage URPs in research studies is still in its infancy [17].

### Goal of This Paper

This paper aims to (1) characterize the socioeconomic and demographic differences among individuals who enrolled and participated in a US-based pregnancy study—the Better Understanding the Metamorphosis of Pregnancy (BUMP) study—through various remote, digital, and traditional recruitment methods and (2) to determine whether social media outreach is an effective means of recruiting and retaining historically URPs to participate in pregnancy-related digital health research.

## Methods

### Overview

The BUMP study is a prospective, observational, cohort study conducted by 4YouandMe, a nonprofit organization that pilots open-source, digital health research. The BUMP study aims to leverage DHTs in the collection of objective and subjective measurements of health to increase understanding of pregnancy and subsequent complications in a sample of women, aged 18-40 years, in the United States (n=524) [29]. The BUMP study sample was primarily White non-Hispanic (381/524, 72.7%) and between 26-35 years of age (363/524, 69.3%). The first participant was enrolled in the BUMP study on February 23, 2021, and the last participant completed the study on July 1, 2023. Participants were enrolled over a 6- to 12-month period and followed for up to 16 months.

The BUMP study used various recruitment methods, including a patient portal for genetic testing results during pregnancy, social media platforms like Facebook, Reddit, and Instagram through both paid and unpaid advertisements; in-person clinics at a trusted community health center offering prenatal and postnatal care; as well as other outreach strategies such as word of mouth and participant referrals. Data were collected through surveys and active tasks on a study smartphone app, wearable DHTs (eg, a Garmin smartwatch, an Oura smart ring, and a Bodyport smart scale), and patients’ electronic medical records.

### Recruitment

**Figure 1:**
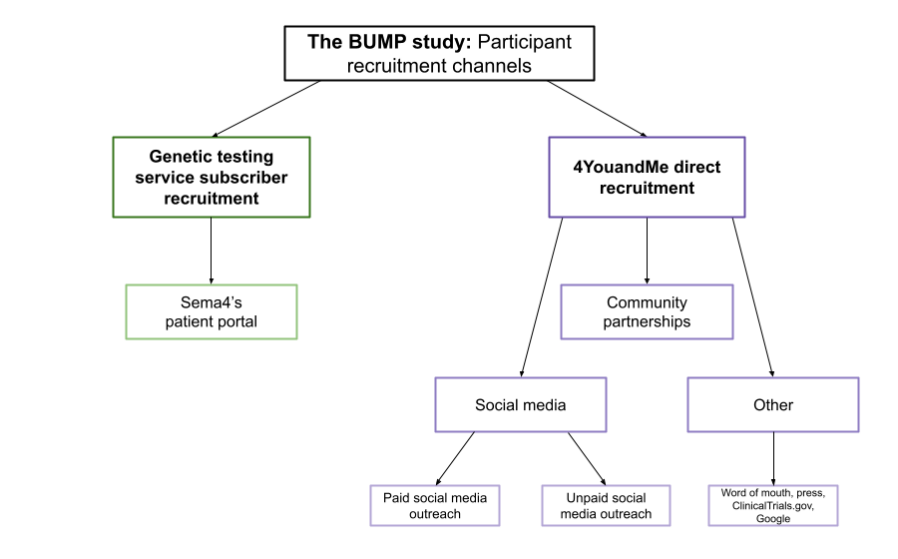
Flowchart of recruitment channels for the Better Understanding the Metamorphosis of Pregnancy (BUMP) study.

Recruitment for the BUMP study was primarily conducted via 2 methods: genetic testing service subscriber recruitment (Sema4) and 4YouandMe direct recruitment. Genetic testing service subscriber recruitment includes outreach to subscribers to genetic testing via Sema4’s patient portal. 4YouandMe direct recruitment consists of 3 recruitment suboutlets: social media (including both paid and unpaid outreach), community partnerships, and others (eg, word of mouth, press, and ClinicalTrials.gov; Figure 1).

#### Genetic Testing Service Subscriber Recruitment (Sema4)

Primary recruitment for the BUMP study was completed via Sema4’s patient portal, specifically focusing on those who signed up to receive results of genetic testing results during pregnancy. Sema4’s patient portal combines mobile health and eHealth as it provides a mechanism for Sema4 to engage with its patients via communication of test results and management of electronic consents [29]. Patients who consented to having their electronic medical record data accessed by Sema4, and those requesting results of genetic testing or other tests commonly associated with pregnancy, received an email from Sema4 with information about the BUMP study, including contact information for study staff if further interested in participation [29].

#### 4YouandMe Direct Recruitment (Social Media Recruitment, Community Partnerships, and Others)

All recruitment conducted by methods other than outreach to genetic testing service subscribers via Sema4’s patient portal of genetic testing subscribers are referred to as 4YouandMe direct recruitment. 4YouandMe direct recruitment was conducted primarily through social media platforms (eg, Facebook, Reddit, and Instagram).

#### Unpaid Social Media Outreach (Social Media Recruitment)

Social media sites with moderated group forums related to pregnancy (eg, Facebook and Reddit) were the focus of unpaid social media recruitment efforts to reach the wider population of all study-eligible pregnant women for the BUMP study. The BUMP study staff conducted outreach efforts with forum and group moderators to gain permission to post fliers on these forums. The BUMP study staff monitored public response to the live group and forum posts and interacted with moderators and interested individuals by answering questions and engaging with posted content. The goal was to build trust and credibility within these online communities while mitigating some of the noted common challenges faced in social media–based recruitment.

Text study descriptions and image-heavy digital recruitment fliers were used to reach potential participants and posted as both standalone and response posts in various pregnancy-related online forums (eg, “Pregnant Women’s Group” and “Mom’s Talk: Pregnancy to Toddler” on Facebook; and “Science Based Parenting,” “Pregnant,” “Mommit,” and monthly “BUMP Groups,” ie, “January 2023 BUMP Group” on Reddit; Multimedia Appendices 1-4).

#### Paid Social Media Outreach (Social Media Recruitment)

A digital recruitment flier was posted on 4YouandMe’s personal Instagram profile, and its visibility was boosted via Instagram’s paid promotion feature, which allows you to select a “goal” (ie, driving traffic to a specific website). The total spend for paid social media recruitment was US $1939.30 over several paid Instagram advertisements.

Interested participants in the BUMP study were able to click on the advertisement and were then guided directly to complete the study interest form, hosted on the secure electronic data capture platform, REDCap (Research Electronic Data Capture; Vanderbilt University). The advertisement parameters for the paid social media outreach method included women, aged 18-42 years, currently living in the United States; and women, aged 18-36 years, living in the lowest 10% of United States zip codes by per capita income.

#### Community-Based Recruitment or Outreach (Community Partnerships)

Community-based partnerships were formed with the Moses/Weitzman Health System, a large federally qualified health center that works to deliver comprehensive health care services to those in need throughout the state of Connecticut. Partnership costs were covered by an agreed-upon fee for collaboration between Moses/Weitzman Health System and 4YouandMe. Medical assistants at three locations (Meriden, Middletown, and Clinton) that provide prenatal and postnatal care were educated by the BUMP study staff on the study overview, goals, eligibility requirements, wearable DHT use, and participant expectations. Moses/Weitzman Health System staff members were also provided with printed recruitment materials for the BUMP study.

Eligible potential participants were informed about the BUMP study by Moses/Weitzman Health System staff during one of their initial prenatal visits. Potential participants who expressed interest in the BUMP study were given a printed flier, tailored to the outreach of this specific community, with a scannable QR code linking interested participants to the study interest form, as well as contact information for study staff available to answer any questions before providing any personal information (Multimedia Appendices 5-6).

#### Other Recruitment Sources (Other)

Additional recruitment for the 4YouandMe direct outreach method was completed by other avenues such as referrals from current BUMP study participants to friends, family, and colleagues; general word of mouth about the BUMP study; articles published about 4YouandMe and the BUMP study on the Oura Ring blog and in STAT Magazine; and other sources such as Google and ClinicalTrials.gov.

### Statistical Analysis

Engagement, for the purpose of this analysis, is defined as any interaction by a potential participant with the BUMP study material such as clicking on an advertisement or filling out a study interest form. Engagement with study advertisements from social media sites was tracked by Google Analytics, and REDCap was used to monitor completed study interest forms and collect additional information regarding recruitment channels. Due to the nature of recruitment conducted of genetic testing service subscribers via Sema4’s patient portal, engagement was unable to be calculated for this group. Enrollment is defined as any participant who signed the informed consent and officially enrolled into the study, for any duration of time. In contrast, retention refers to participants who completed the full study period through birth or at least 9 months.

Engagement, enrollment, and retention rates were stratified by different recruitment channels and race. Sociodemographic characteristics including age, race or ethnicity, and income per capita (US $) by zip code were compared across different recruitment channels. Enrolled participants were also asked to share their home zip code upon enrollment. Participant’s zip codes were used to calculate the distribution of per capita income of enrolled study participants, stratified by their recruitment method. Income data were provided by the United States Census Bureau’s 2020 American Community Survey [30]. All analyses were conducted using Microsoft Excel (version 16.0).

### Ethical Considerations

Human subjects research for the BUMP study was conducted in accordance with all applicable ethical guidelines and received full approval from the institutional review board, Advarra (Pro00047893). Participant confidentiality was maintained via deidentified data, secure data storage, limiting access of identifiable information to authorized members of the research team, and following clearly outlined privacy measures in the electronic consent (eConsent) document.

The BUMP study app served as the participants’ hub for all data collection and study-related communication. Participants were instructed to download the app from the App Store (Apple Inc) or Google Play Store (Android). After downloading the study app, participants were walked through the study onboarding process, beginning with completing an eConsent form. ResearchKit’s eConsent framework was used (for iOS) that includes brief summary screens with a link out to a “learn more” section to convey the consent narrative in a more digestible way. Participants were then provided with the eConsent in its entirety for their review and given a brief comprehension quiz before digitally signing the eConsent form. Participants immediately receive a digital, signed copy of the eConsent to their provided email address [29].

BUMP study participants were financially compensated in accordance with their engagement in daily surveys and active task completions on the BUMP study app. Participants received daily reward points that were tallied at the end of the study, and financial compensation was awarded proportionate to their accumulated study points for task completion. Participants were able to earn up to US $25 per month of participation in the BUMP study, for up to 12 months (including the 9-month study period and 3-month postpartum observation period). Participants who completed the study were also offered to keep their wearable devices—the Oura smart ring and Garmin smartwatch.

Further details of the BUMP study are described in external publications [29].

## Results

### Engagement, Enrollment, and Retention

Across all recruitment channels, 524 pregnant participants were enrolled in the BUMP study: 379 (72.3%) completed the full study period, 384 (73.3%) participants were recruited via Sema4’s portal for genetic testing service subscribers, and 140 (26.7%) participants were recruited to 4YouandMe directly from the other recruitment approaches. Participants recruited via Sema4’s patient portal (genetic testing service subscribers) had similar rates of study retention as those recruited via unpaid social media (Reddit and Facebook) and paid social media (Instagram). The Moses/Weitzman Health System had the lowest percentage of study retention.

**Figure 2:**
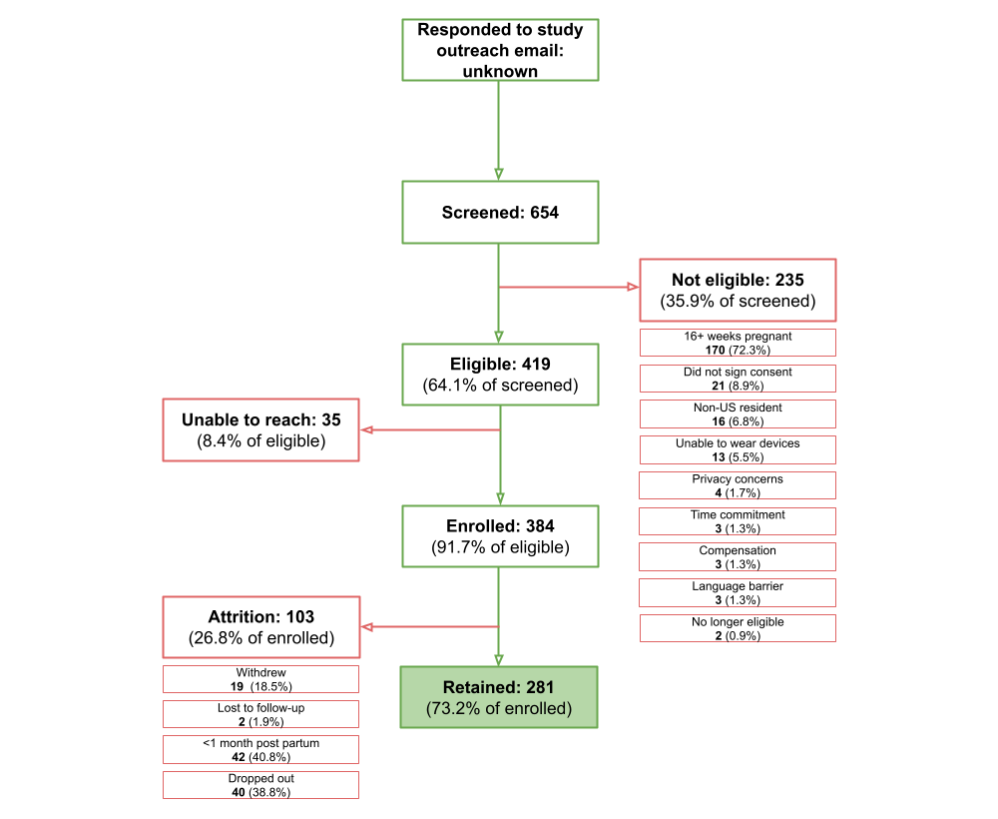
Engagement, enrollment, and retention for the Better Understanding the Metamorphosis of Pregnancy (BUMP) study from outreach via genetic testing service subscribers (Sema4).

Genetic testing service subscriber recruitment was conducted via Sema4’s patient portal for genetic testing service subscribers. Engagement data for this group is unknown. Of those who responded to an initial email with information about the BUMP study, 654 participants completed a screening call with a study coordinator. Of this group, 64.1% (419/654) met eligibility criteria, and 91.7% (384/654) of eligible participants enrolled in the study, resulting in 73.2% (281/654) of participants retained for the full study. (Figure 2).

**Figure 3:**
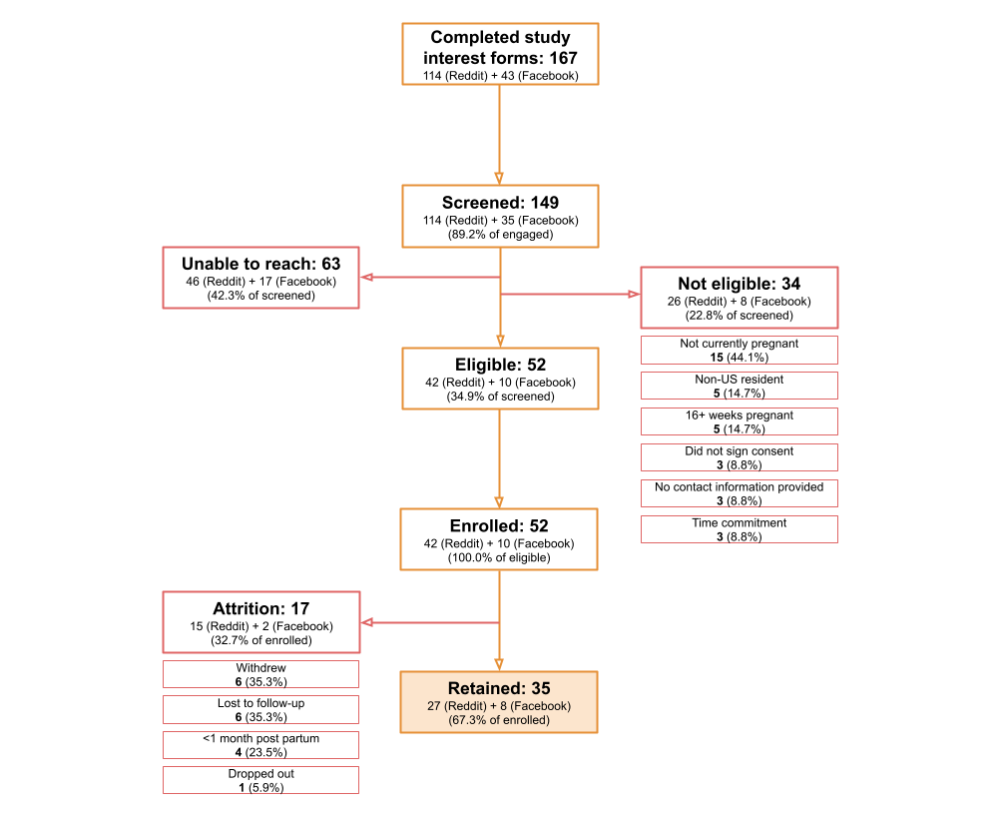
Engagement, enrollment, and retention for the Better Understanding the Metamorphosis of Pregnancy (BUMP) study from outreach via unpaid social media recruitment (Reddit and Facebook).

Social media recruitment of participants was conducted via unpaid advertisements (Reddit and Facebook) and paid advertisements (Instagram). Of those who engaged with an unpaid ad posted on Reddit and Facebook, 74.9% (167/223) completed a study interest form. Of this group, 31.1% (52/167) met eligibility criteria, and 100% (52/52) of eligible participants enrolled in the study, resulting in 67.3% (35/52) retained by the study’s end (Figure 3).

**Figure 4:**
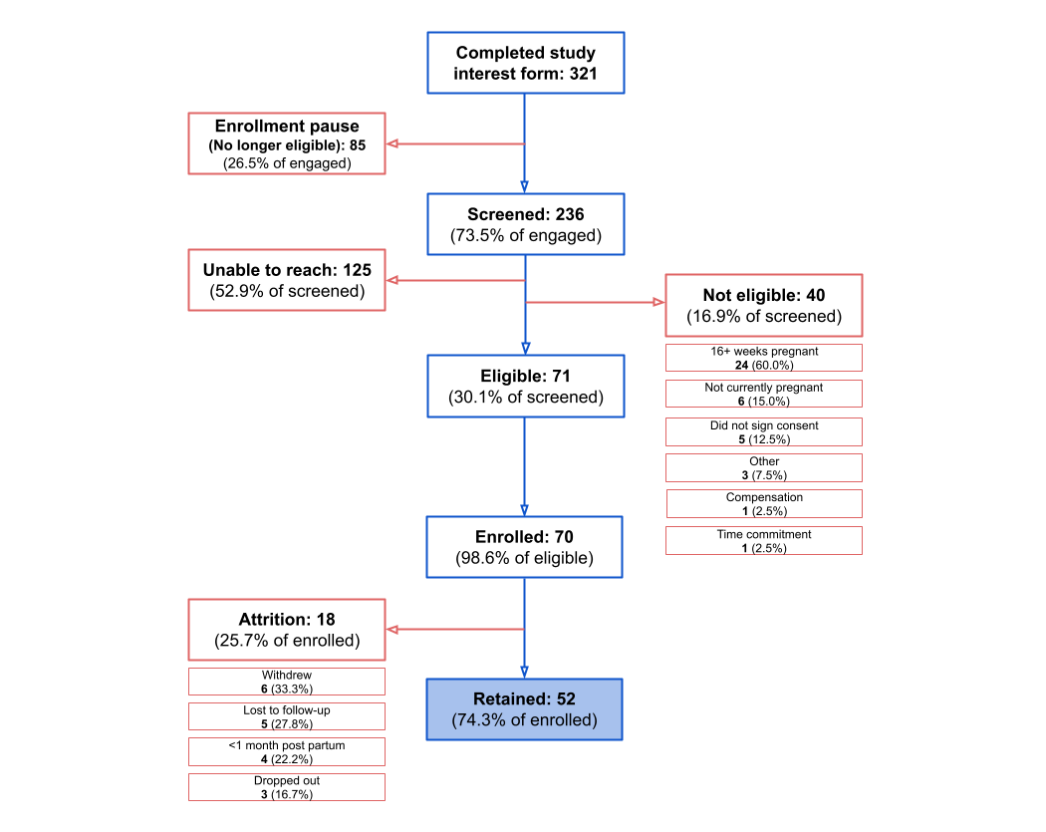
Engagement, enrollment, and retention for the Better Understanding the Metamorphosis of Pregnancy (BUMP) study from outreach via paid social media recruitment (Instagram).

For individuals recruited from paid social media advertisements on Instagram, 321 individuals completed study interest forms; of those, 85 (26.5%) were not eligible for enrollment as a result of a studywide enrollment pause due to high recruitment volume. These individuals were notified of the enrollment pause but were not recontacted for further enrollment. Of the remaining 71 eligible participants, 70 (98.6%) enrolled in the study, and 74.3% (52/70) of those enrolled were retained (Figure 4).

**Figure 5:**
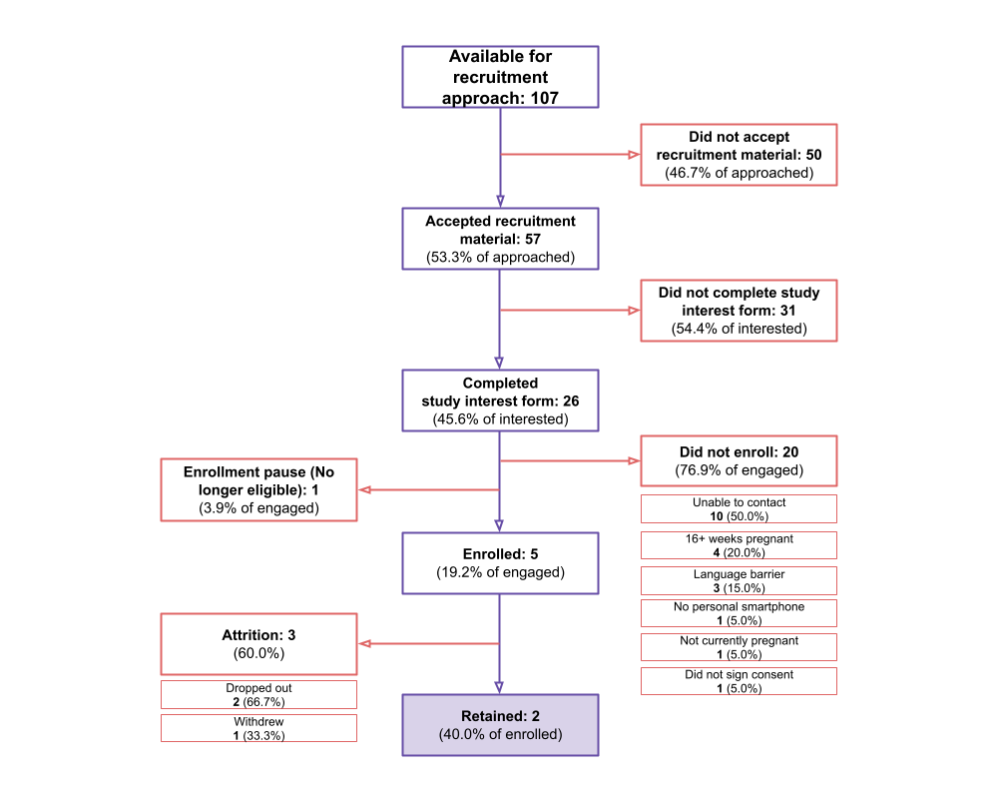
Engagement, enrollment, and retention for the Better Understanding the Metamorphosis of Pregnancy (BUMP) study from outreach via the Moses/Weitzman Health System.

Of participants recruited via in-person visits with their clinician at a community health center providing affordable prenatal care (Moses/Weitzman Health System), 107 met eligibility criteria and were able to be approached on-site by staff. Of those approached, 24.3% (26/107) engaged with study material by completing the study interest form. A total of 76.9% (20/26) of participants did not enroll for a variety of reasons, such as being unable to contact (n=10), having more than 16 weeks pregnant (n=4), language barrier (n=3: n=2 Spanish and n=1 Haitian Creole), having no personal smartphone (n=1), not currently pregnant (n=1), or did not sign the consent (n=1). In total, 1 participant was no longer eligible due to the previously mentioned studywide enrollment pause, 5 participants ultimately enrolled in the study, and 40% (2/5) were retained—the lowest retention rate of any used recruitment method (Figure 5).

Over a 25-week period of active advertisements on both paid and unpaid social media channels (excluding community partnerships and other methods), 491 study interest forms were completed (the total from all 4YouandMe direct recruitment outlets was 594). This resulted in 122 enrolled participants (the total from 4YouandMe direct recruitment was 140) via paid and unpaid social media recruitment into the BUMP study (Table 1).

**Table 1:** Engagement and enrollment characteristics across different social media channels.

	Unpaid advertisements (Reddit)	Unpaid advertisements (Facebook)	Paid advertisements (Instagram)
Ad reach, n	—^a^	—	97,475
Website or advertisement taps, n	197	26	1534
Completed study interest forms, n	124	43	324
Participants enrolled, n	42	10	70
Start and end dates	April 5, 2022, to September 8, 2022	May 18, 2022, to September 8, 2022	September 2, 2022, to October 19, 2022

^a^Not applicable.

### Characteristics of the Cohort

The majority of enrolled participants in the BUMP study were between the ages of 26-35 years (363/524, 69.3%) and White non-Hispanic (381/524, 72.7%; Table 2). For those that engaged with materials via 4YouandMe direct recruitment, participants were asked “Where did you hear about the BUMP study?” and were also given the option to self-identify both their race and ethnicity. Of participants that responded via each recruitment outlet (Reddit: n=124, Moses/Weitzman Health System: n=26, and Instagram: n=321), Reddit had the highest percentage of White non-Hispanic potential participants (98/124, 79%), while the Moses/Weitzman Health System had the lowest percentage of White non-Hispanic potential participants (7/26, 26.9%) and the highest relative percentage of Black potential participants (5/26, 19.2%), followed by Instagram (32/321, 9.9%; Table 3).

**Table 2:** Characteristics of the overall cohort among those who enrolled and were retained in the BUMP^a^ study.

Characteristics			Genetic testing service subscriber recruitment (Sema4)					4YouandMe direct recruitment (social media, community partnerships, and other)					The BUMP study (all recruitment methods)		
			Enrolled (n=384)		Retained (n=281)		Enrolled (n=140)			Retained (n=98)		Enrolled (n=524)			Retained (n=379)
**Age group (years), n (%)**															
	18-25	18 (4.7)		10 (3.6)		3 (2.1)			3 (3.1)		21 (4)			13 (3.4)	
	26-35	251 (65.4)		187 (66.6)		112 (80)			77 (78.6)		363 (69.3)			264 (69.7)	
	36-45	115 (29.9)		84 (29.9)		25 (21.7)			18 (18.4)		140 (26.7)			102 (26.9)	
**Race, n (%)**															
	Black	15 (3.9)		10 (3.6)		6 (4.3)			4 (4.1)		21 (4)			14 (3.7)	
	White (non-Hispanic)	274 (71.4)		213 (75.8)		107 (76.4)			77 (78.6)		381 (72.7)			290 (76.5)	
	White (Hispanic)	4 (1)		1 (0.4)		5 (3.6)			3 (3.1)		9 (1.7)			4 (1.1)	
	Asian	35 (9.1)		26 (9.3)		8 (5.7)			5 (5.1)		43 (8.21)			31 (8.2)	
	More than one race	18 (4.7)		13 (4.6)		7 (5)			4 (4.1)		25 (4.8)			17 (4.5)	
	Unknown or not reported	38 (9.9)		18 (6.4)		7 (5)			5 (5.1)		45 (8.6)			23 (6.1)	
**Ethnicity, n (%)**															
	Hispanic or Latino	12 (3.1)		6 (2.1)		10 (7.1)			6 (6.1)		22 (4.2)			12 (3.2)	
	Not Hispanic or Latino	372 (96.9)		275 (97.9)		128 (91.4)			91 (92.9)		500 (95.4)			366 (96.6)	
	Unknown or not reported	0 (0)		0 (0)		2 (1.4)			1 (1)		2 (0.4)			1 (0.3)	

^a^BUMP: Better Understanding the Metamorphosis of Pregnancy.

**Table 3:** Characteristics of the cohort among those who engaged with recruitment materials, enrolled, and were retained in the BUMP^a^ study by recruitment method^b^.

Characteristics		Genetic testing service subscriber recruitment		4YouandMe direct recruitment														
		Sema4		Instagram				Facebook				Reddit				Moses/Weitzman Health System		
		Enrolled (n=384)	Retained (n=281)	Engaged (n=321)	Enrolled (n=70)	Retained (n=52)	Engaged (n=43)		Enrolled (n=10)	Retained (n=8)	Engaged (n=124)		Enrolled (n=42)	Retained (n=27)	Engaged (n=26)		Enrolled (n=5)	Retained (n=2)
**Age group (years), n (%)**																		
	18-25	18 (4.7)	10 (3.6)	40 (12.5)	1 (1.4)	1 (1.9)	4 (9.3)		1 (10)	1 (12.5)	5 (4)		1 (2.4)	1 (3.7)	8 (30.8)		0 (0)	0 (0)
	26-35	251 (65.4)	187 (66.6)	224 (69.8)	56 (80)	42 (80.8)	35 (81.4)		7 (70)	6 (75)	102 (82.3)		34 (80.9)	20 (74)	16 (61.5)		4 (80)	2 (100)
	36-45	115 (29.9)	84 (29.9)	57 (17.8)	13 (18.6)	9 (17.3)	4 (9.3)		2 (20)	1 (12.5)	17 (13.7)		7 (16.7)	6 (22.2)	2 (7.7)		1 (20)	0 (0)
**Race, n (%)**																		
	Black	15 (3.9)	10 (66.7)	32 (9.9)	2 (2.9)	1 (50)	4 (9.3)		0 (0)	0 (0)	4 (3.2)		1 (2.4)	1 (3.7)	5 (19.2)		1 (20)	1 (50)
	White (non-Hispanic)	274 (71.4)	213 (77.7)	215 (66.9)	52 (74.3)	40 (76.9)	28 (65.1)		9 (90)	7 (87.5)	98 (79)		34 (80.9)	22 (81.5)	7 (26.9)		2 (40)	1 (50)
	White (Hispanic)	4 (1)	1 (25)	18 (5.6)	3 (4.3)	2 (66.7)	2 (4.7)		0 (0)	0 (0)	5 (4)		0 (0)	0 (0)	5 (19.2)		1 (20)	0 (0)
	Asian	35 (9.1)	26 (74.3)	16 (4.9)	4 (4.29)	2 (66.7)	1 (2.3)		0 (0)	0 (0)	9 (7.3)		5 (11.9)	3 (11.1)	0 (0)		0 (0)	0 (0)
	American Indian or Alaska Native	0 (0)	0 (0)	1 (0.3)	0 (0)	0 (0)	1 (2.3)		0 (0)	0 (0)	0 (0)		0 (0)	0 (0)	1 (3.9)		0 (0)	0 (0)
	Native Hawaiian or Other Pacific Islander	0 (0)	0 (0)	0 (0)	0 (0)	0 (0)	1 (2.3)		0 (0)	0 (0)	0 (0)		0 (0)	0 (0)	0 (0)		0 (0)	0 (0)
	More than one race	18 (4.7)	13 (72.2)	18 (5.6)	5 (7.1)	3 (60)	4 (9.3)		0 (0)	0 (0)	6 (4.8)		2 (4.8)	1 (3.7)	2 (7.7)		0 (0)	0 (0)
	Unknown or not reported	38 (9.9)	18 (47.4)	21 (6.5)	5 (7.1)	4 (80)	2 (4.7)		1 (10)	1 (12.5)	2 (1.6)		0 (0)	0 (0)	6 (23)		1 (20)	0 (0)
**Ethnicity, n (%)**																		
	Hispanic or Latino	12 (3.1)	6 (2.1)	36 (11.2)	5 (7.1)	4 (7.7)	4 (9.3)		0 (0)	0 (0)	7 (5.7)		0 (0)	0 (0)	13 (50)		3 (60)	1 (50)
	Not Hispanic or Latino	372 (96.9)	275 (97.9)	276 (85.9)	63 (90)	47 (90.8)	39 (90.7)		10 (100)	8 (100)	114 (91.9)		42 (100)	27 (100)	13 (50)		2 (40)	1 (50)
	Unknown or not reported	0 (0)	0 (0)	9 (2.8)	2 (2.9)	1 (1.9)	0 (0)		0 (0)	0 (0)	3 (2.4)		0 (0)	0 (0)	0 (0)		0 (0)	0 (0)

^a^BUMP: Better Understanding the Metamorphosis of Pregnancy.

^b^Values for participants recruited by other methods are included in Multimedia Appendix 7.

**Figure 6:**
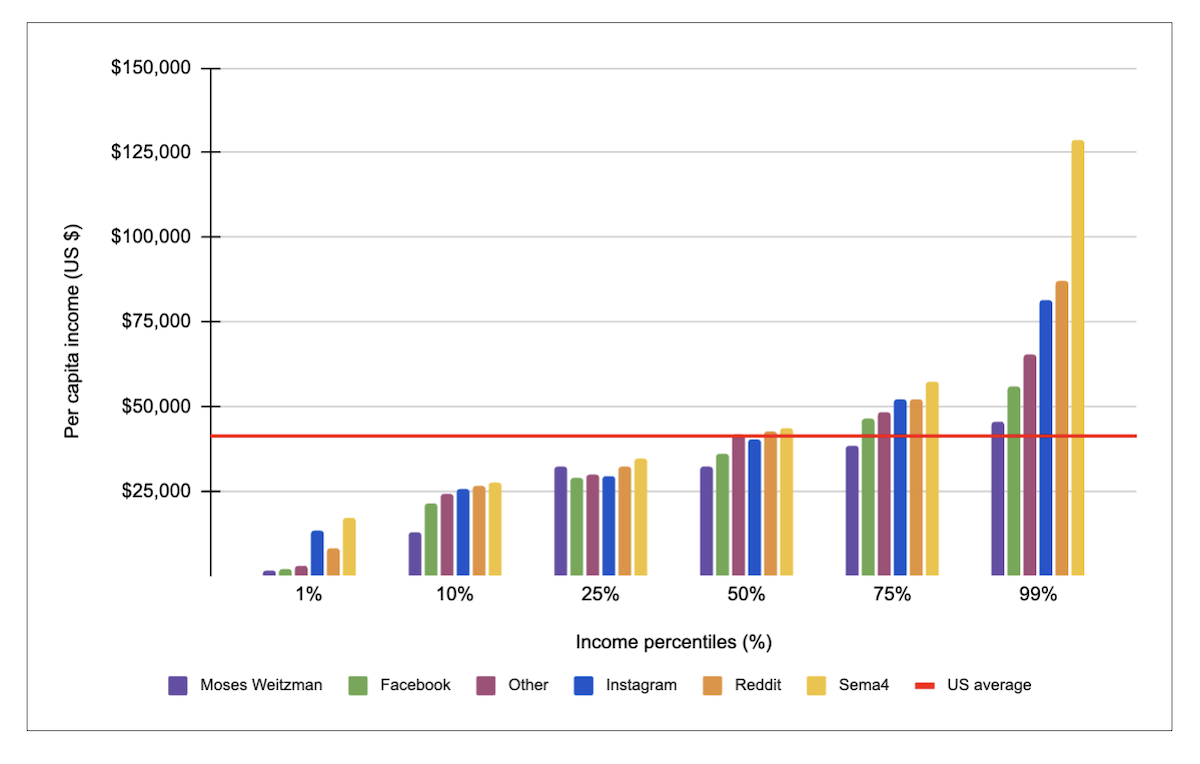
Distribution of income per capita (US $) by zip code of enrolled participants in the Better Understanding the Metamorphosis of Pregnancy (BUMP) study by recruitment method (genetic testing subscriber recruitment: n=384 and 4YouandMe direct recruitment: n=140).

Those recruited via Sema4 (genetic testing service subscribers) had the highest participant income per capita by zip code at nearly every income percentile (Figure 6). Participants recruited via community outreach partnerships (Moses/Weitzman Health System) had the lowest number of participants in the 50-99th percentiles of per capita income by zip code, studywide (Figure 6).

## Discussion

### Principal Results

The results of this analysis suggest that the use of social media (paid and unpaid advertisements) as a tool to engage, enroll, and retain URPs in digital health pregnancy research can be effective. Unpaid social media proved to be successful and low-cost, and achieved the highest engagement-to-enrollment rate among all approaches used by the 4YouandMe direct recruitment channel. However, maintaining successful recruitment on these platforms required significant sustained effort and time from the study team.

Paid social media ads on Instagram also effectively reached and engaged pregnant women as URP in digital health research. Running paid advertisements was a low burden on the study team, in comparison to unpaid advertisements, which required continued monitoring and engagement on behalf of the study team. Paid advertisements incurred a cost over 25 weeks; US $1939.30 was spent on Instagram advertisements, resulting in 70 enrollments, or US $27.70 per enrolled participant. This cost per enrolled participant is generally favorable, as previous systematic reviews of the costs of social media–based participant recruitment ranged from US $0.00 to US $517.00 per participant, compared to US $19.00 to US $777.00 for traditional recruitment methods [31]. Additional studies found a mean cost per participant of US $16.22 for Google ads and between US $13.12 and US $250.00 for other traditional methods. Data on the cost-effectiveness of paid social media ads for recruiting participants to research studies are severely limited [32].

Participants recruited via paid social media (Instagram) showed the highest overall retention rate as 74.3% (52/70) of participants enrolled completed the full study period (Table 3). This suggests that paid social media outreach was more effective in engaging and retaining study participants than other recruitment methods. This outreach method was even more effective than recruiting through genetic testing service subscribers (Sema4), where participants may have had a higher intrinsic motivation to participate and stay in the study due to potential previous interest in genetic testing and related research.

Interactions with participants recruited via Instagram during study onboarding revealed a distinct motivation to engage, enroll, and complete the full study period. Although not statistically quantified, this trend may be linked to specific user behavior within the Instagram-recruited cohort, or the platform’s age demographics aligning closely with the study’s target population. Among participants retained in the BUMP study recruited from Instagram, 80.8% (42/52) were aged 26-35 years, compared to 74% (20/27) from Reddit (Table 3). This aligns with broader trends, as 28.3% of the US Instagram users in 2024 were aged 25-34 years, while 44% of Reddit users in 2023 were aged 18-29 years [33,34]. Given that the mean age of first-time mothers in the United States is 27.4 years, Instagram’s higher retention may reflect its unique demographic fit with the specific study population and subsequent increased study interest and engagement [35]. These observations remain speculative, as the available data cannot fully confirm the underlying reasons.

In addition, paid social media recruitment facilitated the widespread engagement and enrollment of URPs. Paid recruitment had a higher proportion of non-White, non-Hispanic respondents who engaged with and enrolled in the BUMP study, in comparison to unpaid social media. Retention of non-White, non-Hispanic participants was low across all recruitment methods.

Recruitment via social media (including paid and unpaid recruitment) also provided access to and enrollment of a different socioeconomic population than those enrolled via genetic testing service subscriber (Sema4) recruitment. Participants recruited via genetic testing service subscribers (Sema4), which is associated with Mount Sinai Health System, reflect a unique group of individuals with means and access to genetic testing. This was shown in the enrolled participants from this group who had significantly higher per capita incomes, exceeding US $75,000 far surpassing the US national average income per capita [36].

In contrast, enrolled participants from social media recruitment and community-based outreach’s highest average per capita incomes were US $74,771.15 and US $45,401.00, respectively (Figure 6). While data are limited, one study suggests that 46% of Instagram users and 23% of Reddit users have a reported household income under US $70,000.00 [37]. From this, we may infer that recruitment via social media (and community partnerships) also facilitated the increased enrollment of socioeconomic-based URPs.

Community-based partnerships for BUMP study recruitment revealed unique and informative data trends. Over a 24-week period at three Moses/Weitzman Health System clinics that serve a large URP, only 53.3% (57/107) accepted recruitment materials (Figure 5). The overall number of potential in-clinic approaches was limited by multiple factors. Time constraints with in-clinic visits and frequent no-shows hindered clinic staff from fully introducing the study to potential participants, thoroughly answering questions, or handing out recruitment material—further illustrating additional downsides of more traditional, in-person recruitment.

In addition, a significant number of potential participants were immediately excluded before the recruitment approach due to a Spanish language barrier, curtailing the overall number of eligible approaches. Of those remaining participants eligible for approach, some displayed immediate apprehension to study engagement upon simply hearing the word “study,” and one participant explicitly reported “not wanting to feel like a guinea pig” to clinic staff.” These responses show an intrinsic hesitancy to engage in research studies and a deep mistrust of research overall, even when the information is conveyed by familiar health care providers or their staff.

Community health center–recruited participants who indicated study interest also showcased population-specific barriers to study enrollment or participation. Despite accepting recruitment material, 15% (3/20) of participants did not express sufficient English language fluency to complete surveys and tasks entirely in English on the study app. A total of 50% (10/20) of participants who engaged with study materials were difficult to reach for a further onboarding call (Figure 5). While many of these participants were responsive via text message, BUMP study staff reported difficulty completing study onboarding via phone with participants, citing their extensive and limiting work schedules. Other significant, population-specific barriers to study enrollment included those who did not own a personal smartphone.

Despite dedicated efforts from the BUMP study team and Moses/Weitzman Health System staff, recruiting, engaging, and enrolling participants from community health centers proved uniquely challenging compared to other methods. Of the potential participants who received recruitment materials, only 8.8% (5/57) ultimately enrolled in the study (Figure 5). This low enrollment underscores many of the well-documented challenges of maintaining consistent enrollment among URPs when relying solely on community outreach channels. It also highlights opportunities for overcoming these challenges in future studies.

Strategies to address logistical and participant-voiced barriers may include conducting digital recruitment follow-ups with no-show potential participants, enhancing language inclusivity in recruitment and study app materials, incorporating text messaging for primary communication, providing smartphones for study participant use, and embedding opportunities for recruitment outreach into clinical care. Seamless communication between researchers, providers, and medical staff can also aid in addressing participants’ needs in real time as researchers work with clinic staff, using feedback from potential participants, to address population-specific concerns and develop tailored language and approaches; combating mistrust head-on by leading with transparency; and creating a culture of embedding options for research participation into clinical care.

### Limitations

Due to the methodology of recruitment and outreach for the BUMP study via genetic testing service subscribers (Sema4), overall study interest and engagement were unable to be followed and analyzed in the same manner as 4YouandMe direct recruitment avenues. Similarly, demographic information (eg, race or ethnicity, socioeconomic status) was not provided for interested or potential participants from this group, limiting the comparison and further analysis of engagement with study materials by various URPs between recruitment of genetic testing service subscribers and social media and other methods.

Additional limitations were present in the analysis of the recruitment potential for the general pregnant population via social media platforms. We were unable to discern how many of the total reached population was actually pregnant or study eligible; this was particularly relevant for paid social media outreach and recruitment. For paid social media outreach, we relied on inputting the lowest 10% of US zip codes by per capita income to increase our socioeconomic diversity; however, it is unknown if zip codes are too narrow of a parameter for focused Instagram advertisements, ultimately impacting an in-depth review of engagement statistics.

True analysis of study engagement to enrollment statistics was further limited by the presence of a study enrollment pause, due to the high volume of completed study interest forms and potential participants. This enrollment pause rendered 132 participants no longer eligible for study participation. Without this pause, the engagement-to-enrollment rate may have reached 45.8% (272/594). Recruitment for the BUMP study via the various 4YouandMe direct approaches was not conducted for the entire study duration, impacting the overall ability to assess engagement to enrollment effectiveness. Finally, the small size of this study sample (n=524) indicates the need for larger, similar studies to further validate the trends observed.

### Conclusions

Social media and virtual outreach are both emerging and exciting tools that researchers can use to engage and recruit various URPs to participate in digital health research studies, specifically about pregnancy. Paid social media advertisements provide unique, innovative, and low-time-burden opportunities for researchers to engage and interact with a larger volume of URPs in comparison to traditional recruitment methods.

Though commonly perceived as the gold standard for outreach to URPs, community-based partnerships, even when combined with other outreach methods, did not provide a smooth, consistent path to outreach, engagement, or retention of URPs in digital health research. This lower engagement and retention underscore the need for further tailored strategies of addressing additional barriers to sustained participation in digital health studies for URPs to better bridge the digital divide and engage URPs.

By embracing novel recruitment outreach methods such as via social media platforms, researchers have a unique opportunity to refine often stagnant, traditional recruitment strategies and adapt to an evolving digital landscape. The integration of community-driven recruitment approaches with tailored digital interventions that uniquely address the needs of URPs will create more inclusive and equitable opportunities for participation in digital health studies and foster more representative and impactful research that may ultimately improve health outcomes for URPs and others alike.

## Appendix

Multimedia Appendix 1 The BUMP study recruitment flier for outreach via 4YouandMe direct recruitment approaches designed for long-form social media sites (eg, Reddit). BUMP: Better Understanding the Metamorphosis of Pregnancy.

Multimedia Appendix 2 The BUMP study recruitment flier for outreach via 4YouandMe direct recruitment approaches designed for long-form social media sites (eg, Reddit). BUMP: Better Understanding the Metamorphosis of Pregnancy.

Multimedia Appendix 3 The BUMP study recruitment flier for outreach via 4YouandMe direct recruitment approaches designed for long-form social media sites (text version), designed for long-form social media sites with specific forums that did not allow images (eg, Reddit). BUMP: Better Understanding the Metamorphosis of Pregnancy.

Multimedia Appendix 4 The BUMP study recruitment flier for outreach via 4YouandMe direct recruitment approaches designed for long-form social media sites, tailored for image-dominant social media sites (eg, Instagram). BUMP: Better Understanding the Metamorphosis of Pregnancy.

Multimedia Appendix 5 The BUMP study recruitment flier for outreach via 4YouandMe direct recruitment approach, tailored to community health partnerships (Moses/Weitzman Health System). BUMP: Better Understanding the Metamorphosis of Pregnancy.

Multimedia Appendix 6 The BUMP study recruitment flier for outreach via 4YouandMe direct recruitment approach (alternate flier), tailored to community health partnerships (Moses/Weitzman Health System). BUMP: Better Understanding the Metamorphosis of Pregnancy.

Multimedia Appendix 7 Characteristics of the cohort among those who engaged (ENG) with recruitment materials, enrolled (ENR), and were retained (RTD) in the BUMP study for participants recruited via other methods. BUMP: Better Understanding the Metamorphosis of Pregnancy.

## Abbreviations

BUMP: Better Understanding the Metamorphosis of Pregnancy
DHTs: digital health technologies
eConsent: electronic consent
NIH: National Institutes of Health
REDCap: Research Electronic Data Capture
URPs: Underrepresented populations

## Data Availability

The datasets generated or analyzed during this study will be hosted in the public domain and available on the Synapse platform at Sage Bionetworks [29]. Please check the 4YouandMe website or contact the corresponding author for an update on when study data will be available and how to access it.
