# Supplementary figures and images for: Using Social Media to Engage and Enroll Underrepresented Populations: Longitudinal Digital Health Research
Source: JMIR Form Res. 2025 Apr 15;9:e68093. doi: 10.2196/68093 (PMC12041823; doi:10.2196/68093)

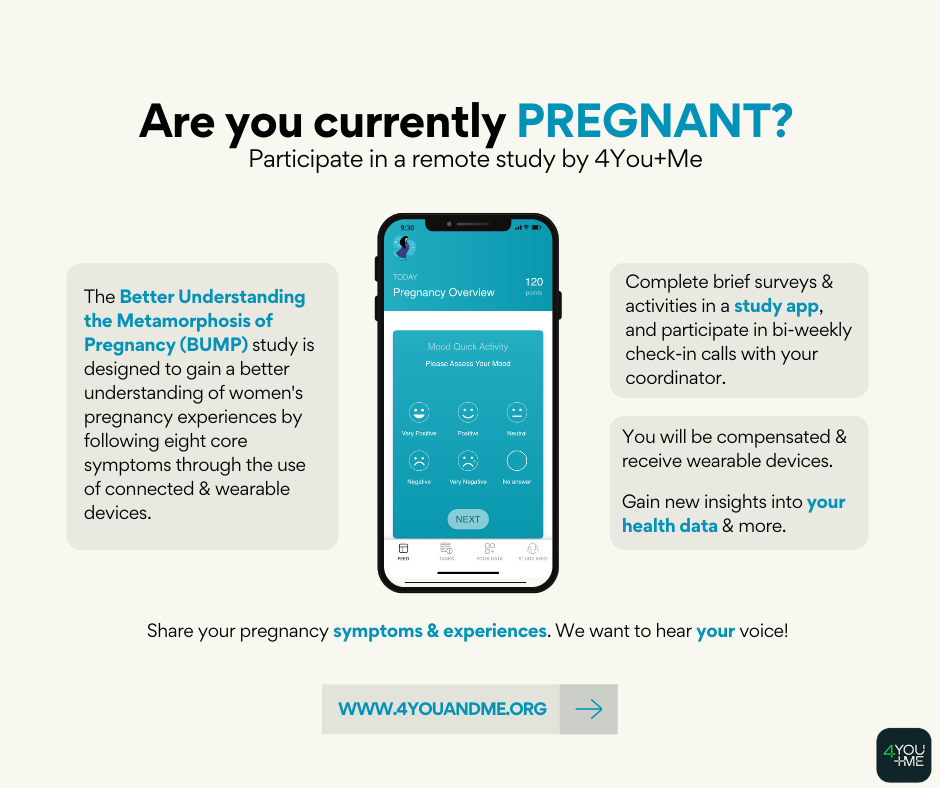

Supplement: Multimedia Appendix 1 [file formative_v9i1e68093_app1.png]

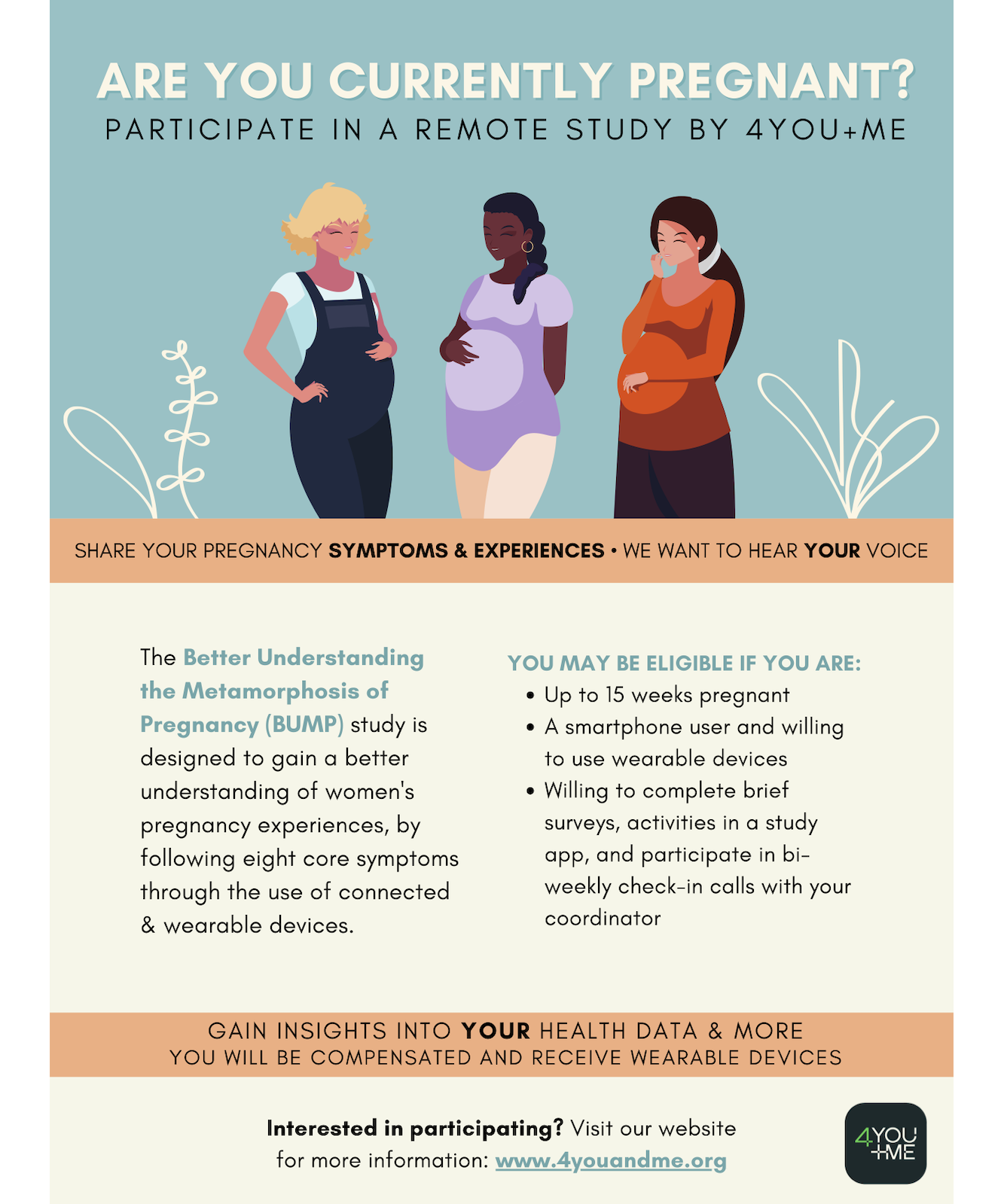

Supplement: Multimedia Appendix 2 [file formative_v9i1e68093_app2.png]

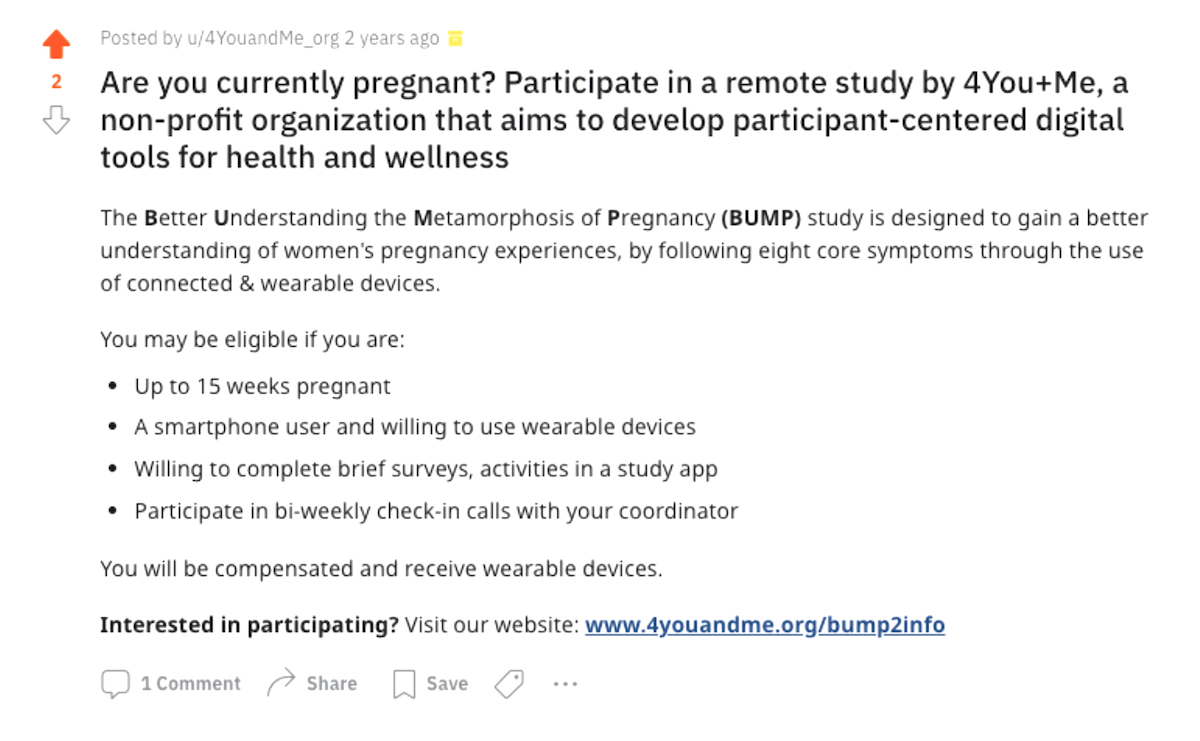

Supplement: Multimedia Appendix 3 [file formative_v9i1e68093_app3.png]

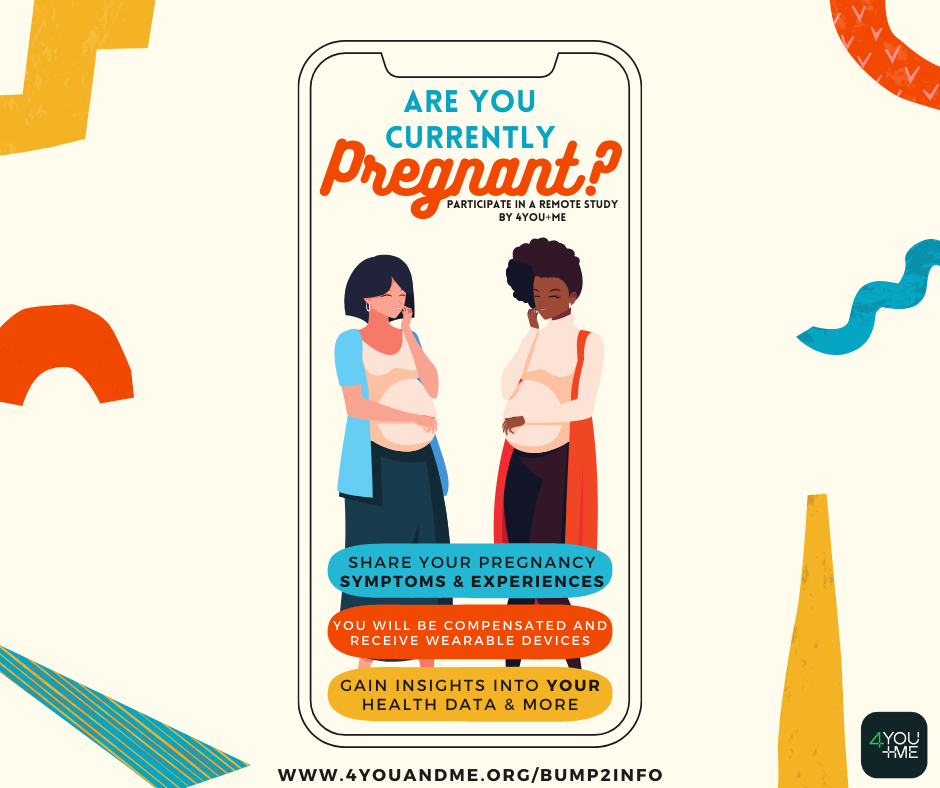

Supplement: Multimedia Appendix 4 [file formative_v9i1e68093_app4.png]

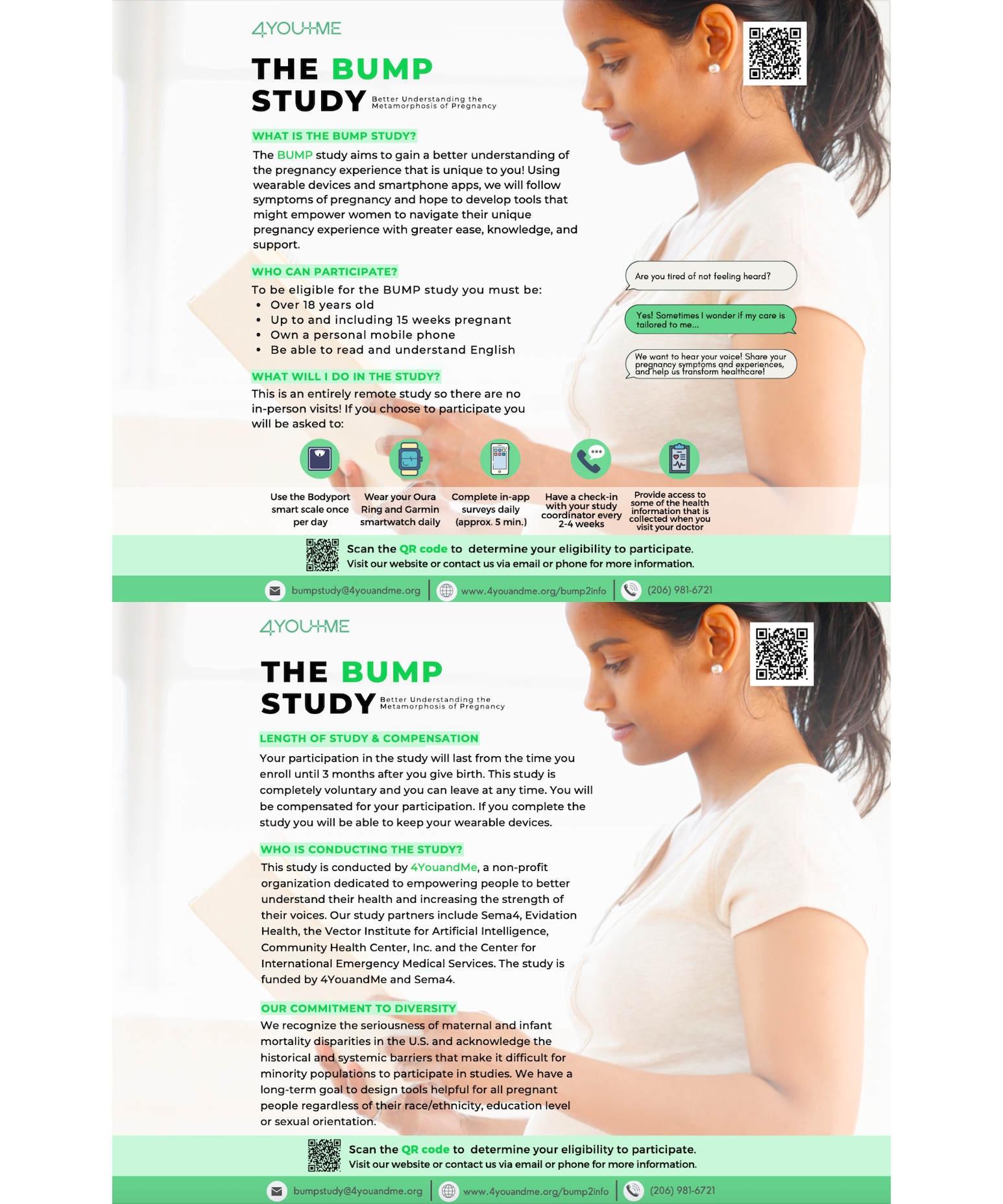

Supplement: Multimedia Appendix 5 [file formative_v9i1e68093_app5.png]

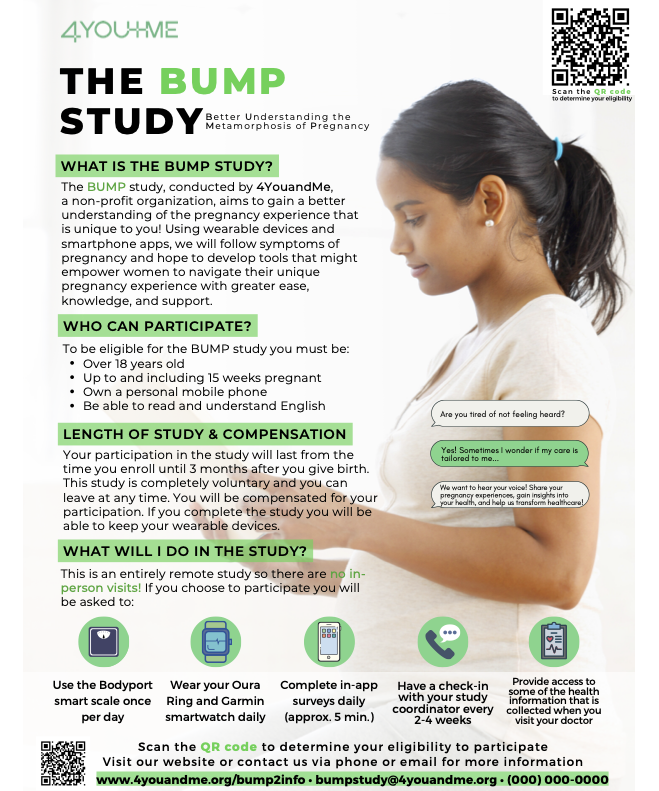

Supplement: Multimedia Appendix 6 [file formative_v9i1e68093_app6.png]

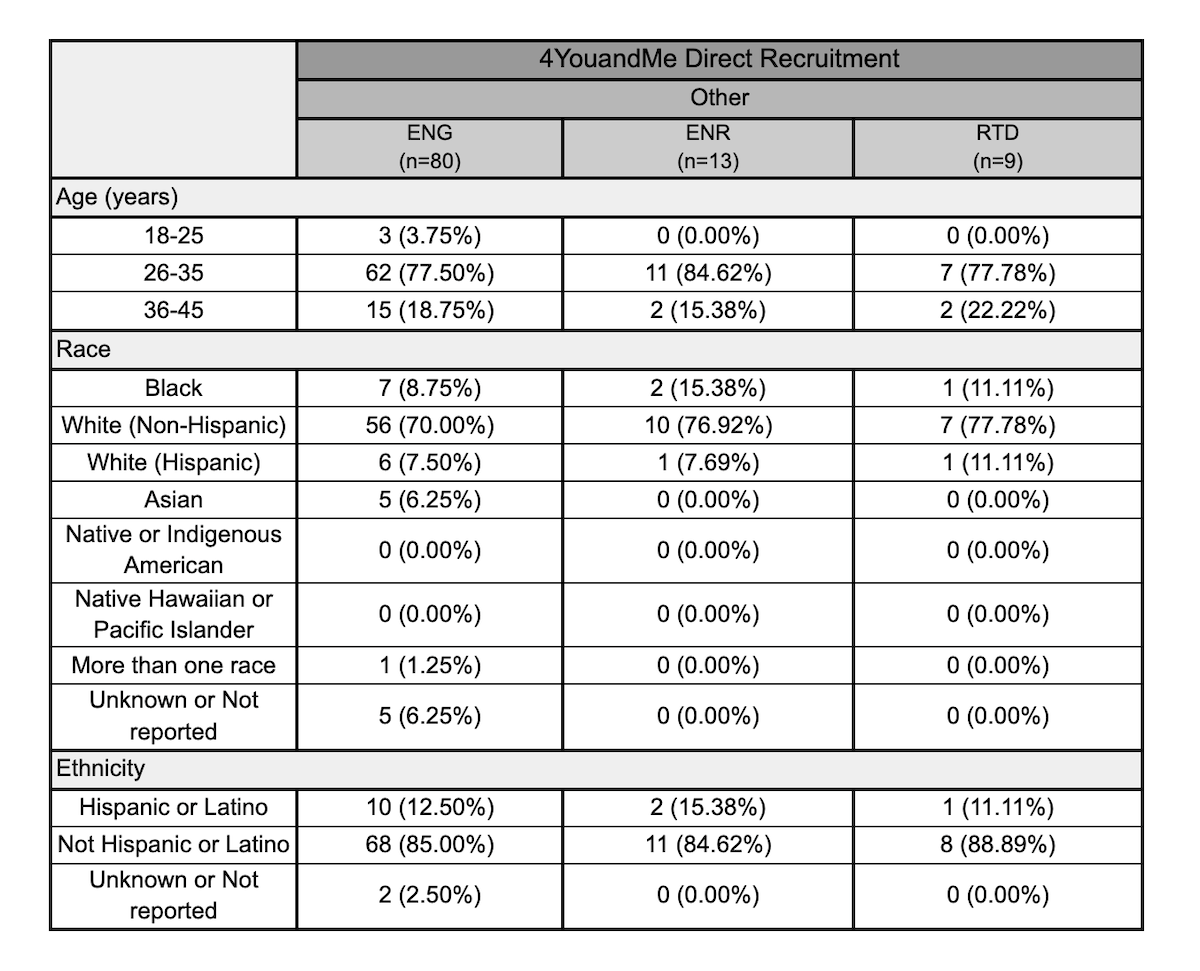

Supplement: Multimedia Appendix 7 [file formative_v9i1e68093_app7.png]
